# The Role of Practitioner Resilience and Mindfulness in Effective Practice: A Practice-Based Feasibility Study

**DOI:** 10.1007/s10488-016-0747-0

**Published:** 2016-07-16

**Authors:** Jo-Ann Pereira, Michael Barkham, Stephen Kellett, David Saxon

**Affiliations:** 1 Department of Psychology, University of Sheffield, Sheffield, UK; 2 Centre for Psychological Services Research, Department of Psychology, University of Sheffield, Sheffield, UK; 3 Centre for Psychological Services Research, Department of Psychology, University of Sheffield; Sheffield Health and Social Care NHS Foundation Trust, Sheffield, UK; 4 Centre for Psychological Services Research, School of Health and Related Research, University of Sheffield, Sheffield, UK

**Keywords:** Therapist effects, Effective practice, Resilience, Mindfulness, Stepped care

## Abstract

A growing body of literature attests to the existence of therapist effects with little explanation of this phenomenon. This study therefore investigated the role of resilience and mindfulness as factors related to practitioner wellbeing and associated effective practice. Data comprised practitioners (n = 37) and their patient outcome data (n = 4980) conducted within a stepped care model of service delivery. Analyses employed benchmarking and multilevel modeling to identify more and less effective practitioners via yoking of therapist factors and nested patient outcomes. A therapist effect of 6.7 % was identified based on patient depression (PHQ-9) outcome scores. More effective practitioners compared to less effective practitioners displayed significantly higher levels of mindfulness as well as resilience and mindfulness combined. Implications for policy, research and practice are discussed.

## Introduction

There is a growing body of evidence that variability exists between psychological therapists in relation to patient outcomes (Baldwin and Imel 2013), a phenomenon termed *therapist effects* (Lutz and Barkham 2015). In general, research studies have reported therapist effects in the region of 5–8 % (e.g., Crits-Christoph et al. 1991; Crits-Christoph and Mintz 1991; Wampold 2001). However, other research has found that therapist effects are minimal, with researchers arguing that the evidence base is actually a methodological artefact (Ehlers et al. 2013; Erickson et al. 2012; Huppert et al. 2014). The most parsimonious explanation for these apparent discrepant views is that therapist effects are manifest only under certain conditions or in specific situations. Factors that could influence the detection of therapist effects include the research paradigm adopted, the sample size of therapists, and patients’ presenting conditions.

In terms of the research paradigm adopted, progress in the investigation of therapist variability has been delayed by the analyses of therapist effects using randomised controlled trials (RCTs; e.g., Clark et al. 2006; Ehlers et al. 2003). Such studies were originally designed as tests of treatment effects rather than therapist effects. Accordingly, research into therapist effects needs to derive from specifically designed studies in which therapists are the primary focus. A corollary of past research has been that studies have employed small numbers of therapists invariably labelled as a fixed variable and thereby limiting the generalizability of findings. In addition, the historical assumption within an RCT is that therapist variability is considered as error rather than as a naturally occurring phenomenon. By contrast, there is an increasing move towards the collection of large routine datasets together with the application of multilevel modeling (MLM) that reflects the hierarchical nature of the data (i.e., patients clustered within therapists) compared to traditional benchmarking analyses (Castonguay et al. 2013). Additionally, there are now statistical guidelines as to the required N of therapists in order to determine the presence of therapist effects (Schiefele et al. 2016). In terms of patient conditions, Saxon and Barkham (2012) found that therapist effects increased according to the severity of patients’ presenting conditions. That is, the more severe a patient’s presenting condition, the more it matters which therapist they see.

In light of these factors, the present study employed a substantial dataset derived from routine practice and applied current multilevel modeling (MLM) as well as traditional benchmarking analytic techniques. It further investigated the role of patient severity in relation to therapist effects.

Notwithstanding establishing the extent of therapist effects, research studies are moving towards building an understanding of what factors account for or lead to variability between therapists and under what conditions (Green et al. 2014; Laska et al. 2013; Nissen-Lie et al. 2013a). Previously, researchers have examined therapists using absolute and distinct variables such as age, race, professional experience, and theoretical orientation. These factors were unlikely to throw any light on the complex dynamics of what practitioners and their patients bring to the helping situation. Following on from successive reviews of therapist variables in the *Handbook of psychotherapy and behavior change* (1971, 1978, 1986, 1994), Beutler et al. (2004) called for research to *“integrate patient, therapist, procedural, and relationship factors”* [p. 292]. In order to meet this goal, it is necessary for research designs to yoke both integrative personal qualities of therapists with measurable patient outcomes.

One therapist quality that has been consistently evidenced has been the role of practitioners’ psychological wellbeing in the therapy situation—a personal quality acknowledged by researchers and patients themselves (e.g., Lafferty et al. 1989; McCarthy and Frieze 1999; Nissen-Lie et al. 2013b). A meta-analysis by Beutler et al. (2004) yielded a positive relationship between practitioner well-being and patient outcome. This finding was consistent irrespective of the heterogeneous nature of patient samples, range of psychological therapies provided, or different treatment formats. In a similar vein, a longitudinal study by Nissen-Lie et al. (2013b) found a direct impact of practitioners’ personal distress on the therapeutic working alliance; patients were particularly sensitive to practitioners’ personal life distress, more so than practitioners themselves and the impact this had on the therapeutic working alliance. Therefore, the typical state of mind that practitioners bring to the therapy situation is an important aspect that possibly explains the variance between observed patient outcomes.

Bajaj and Pande (2015) addressed how resilience and mindfulness play a role in individuals’ wellbeing. The authors used indices of resilience and mindfulness that examined these personal qualities in the context of individuals’ day-to-day living. They argued that individuals develop more resilience as a function of being mindful, which, in turn, contributes to higher levels of wellbeing. Additionally, resilience and mindfulness have been found to contribute towards patient improvement. Green et al. (2014) found that more effective practitioners as compared to less effective practitioners were significantly more resilient. Similarly, more mindful practitioners have been found to yield significantly better patient outcomes (Grepmair et al. 2007; Ryan et al. 2012). Accordingly, resilience and mindfulness might have a specific role to play both in protecting the wellbeing of therapists and yielding better outcomes for their patients.

Resilience has been defined as that which “embodies the personal qualities that enable one to thrive in the face of adversity” (Connor and Davidson 2003; p. 76), while mindfulness refers to “a state of psychological freedom that occurs when attention remains quiet and limber, without attachment to any particular point of view” (Martin 1997; p. 291). The current study conceptualised resilience and mindfulness as personal aspects of practitioners that permeate their daily lifestyle and examined the personal aspects of resilience and mindfulness—alone and in combination—of practitioners who consistently displayed either more effective or less effective practice.

In order to investigate the role of practitioners’ resilience and mindfulness in routine practice, we employed a sample of practitioners employed within the UK’s Improving Access to Psychological Therapies (IAPT) initiative (Clark 2011). IAPT services are commissioned to deliver treatments based upon national clinical guidelines for depression and anxiety (NICE; National Institute for Health and Clinical Excellence 2009, 2011) via stepped care service models. Stepped care configures psychological services via low intensity (i.e., brief, effective and less restrictive) interventions being delivered first. Systematic monitoring of outcomes enables patients to be ‘stepped-up’ to high intensity (i.e., effective, but more intensive and lengthy) interventions according to patients’ needs and responses to treatment (Bower and Gilbody 2005). In IAPT, Step 1 involves contact with a general practitioner for assessment, advice and medication, Step 2 delivers the low intensity interventions (e.g., guided self-help) delivered by psychological wellbeing practitioners (PWPs), and Step 3 delivers high-intensity psychological therapies comprising cognitive-behavior therapy, and counseling. IAPT services aspire to meet a targeted recovery rate of 50 % for their patients (Clark 2011). In reality, however, there is evidence of wide variability between services with a range from 23.9 to 56.5 % being reported (Gyani et al. 2013).

Against this background, the focus of the present study was on the role of resilience and mindfulness, alone and combined, in relation to the delivery of more and less effective practice with patients presenting with anxiety and depression within a single organization providing a stepped care (IAPT) model of delivery. In so doing, we adopted a research paradigm consistent with Beutler et al.’s (2004) call that tested the feasibility of yoking therapists’ personal aspects with their respective effectiveness levels based on patient outcomes in order to determine their contribution towards patient outcomes.

## Method

### Design

The study comprised two datasets: (1) responses provided by practitioners who volunteered to participate in the study, and (2) patient data of the same participating practitioners extracted from a historical patient dataset (spanning 3.4 years; 2010–2013). The patient data was anonymized and based on the IAPT service’s mandatory routine outcome data collection (National IAPT Programme Team 2011). Ethical approval was given by the UK NHS Health Research Authority (reference number: 13/EM/0387).

### Study Sample

#### Practitioners

A total of 115 practitioners were approached to participate with 42 practitioners (36.5 %) volunteering. Across the different practitioner roles, the approximate response rates were: PWPs, n = 11/50 (22.0 %), CBT therapists, n = 12/33 (36.4 %), and counselors, n = 19/32 (59.4 %). Out of these 42 practitioners, 37 had patient data that could be yoked to their personal aspect data. The sample of 37 practitioners was examined against the full IAPT dataset sample of practitioners using multilevel modeling and found to comprise a majority of practitioners who were either effective or more effective within the full practitioner sample (i.e., practitioners who were less effective were less likely to volunteer for the study).

Table 1 summarises the demographic information of the 37 practitioners. The final sample comprised 8 PWPs (21.6 %), 12 CBT therapists (32.4 %), and 17 counselors (45.9 %). Ages ranged from 28 to 72 years with a mean of 47.9 years (SD = 11.9 years). Mean ages were significantly different across the practitioner groups (F(2, 31) = 18.51, *p* = .000), with counselors being significantly older than PWPs (*t*(21) = −6.57, *p* = .000; counselors M = 56.4, SD = 7.2; PWPs M = 34.7, SD = 7.4) and CBT therapists (*t*(25) = −3.76, *p* = .001; counselors M = 56.4, SD = 7.2; CBT therapists M = 43.9. SD = 10.1). Thirty-one practitioners provided information on current working hours that ranged from 15 to 39 h per week, with a mean of 29.9 h (SD = 8.0). Most practitioners were female (75.7 %) and of white ethnicity (97.3 %). Practitioner experience ranged from 0 to 30 years with most practitioners (56.8 %) indicating 0–10 years of full-time equivalent work-related experience. Previous experience comprised a wide range of voluntary and therapeutic roles (e.g., volunteer work with substance misuse patients, GP practice counseling, and employment as a mental health worker). All practitioners were formally trained and qualified. IAPT training of practitioners varied in intensity, in line with the degree of expertise called on from practitioners when treating more or less severely depressed patients. The curriculum for PWP training comprises 4 modules over a period of 45 days (Department of Health 2008a). High intensity training, in comparison, consists of a 1-year full-time course (Department of Health 2008b). Practitioners received regular clinical supervision consistent with their treatment modality.

**Table 1:** Practitioner descriptives

	PWPs (n = 8)		CBT therapists (n = 12)		Counselors (n = 17)		All practitioners (n = 37)	
	M	SD	M	SD	M	SD	M	SD
Age	34.7	7.4	43.9	10.1	56.4	7.2	47.9	11.9
Current working hours (per week)	31.9	7.4	35.5	3.1	23.7	7.4	29.9	8.0
History of number of work-related roles	3.3	1.6	2.6	1.4	5.2	2.2	3.9	2.2

	n	%	n	%	n	%	n	%
Sex								
Male	1	12.5	5	41.7	3	17.6	9	24.3
Female	7	87.5	7	58.3	14	82.4	28	75.7
Ethnicity								
White	8	100.0	12	100.0	16	94.1	36	97.3
Black	0	–	0	–	1	5.9	1	2.7
Practitioner qualification								
Graduate	–	75.0	0	0.0	1	5.9	1	2.7
Post graduate	6		12	100.0	12	70.6	30	81.1
PhD	–		0	0.0	1	5.9	1	2.7
Practitioner work-related experience (WTE bands)								
0–10 years	5	62.5	9	75.0	7	41.2	21	56.8
10–20 years	2	25.1	1	8.3	5	29.4	8	21.6
Over 20 years	1	12.5	2	16.7	5	29.4	8	21.6

#### Patients

The patient study sample that was yoked to the 37 practitioners comprised 4980 patients yielding a mean of 134.6 patients per practitioner (SD = 100.1) and a minimum of 24 patients per practitioner. CBT therapists treated an average of 100 patients, counselors 96 patients, and PWPs 296 patients. This workload is consistent with the job requirements of PWPs at Step 2, which is brief, uses a least restrictive applied treatment intervention, and is characterised as ‘low contact-high volume’. By contrast, ‘high contact-low volume’ treatment is provided by high intensity treatment practitioners (Firth et al. 2015). In terms of the initial severity of depression for patients seeing the three professional roles, the average pre-treatment PHQ-9 scores for PWPs, CBT therapists, and counselors were 15.15 (SD = 5.70), 16.30 (SD = 5.60), and 15.43 (SD = 5.68) respectively. Table 2 presents descriptives of all patients yoked to the 37 practitioners and patients seen by each respective professional role. On scrutiny of initial patient severity levels, relative differences between the professional roles were noted for the treatment of patients with mild and severe depression. CBT therapists treated a comparably lower proportion of patients with mild depression (14.3 %) compared to the mean proportion across all practitioners (18 %), PWPs (19.6 %), and counselors (18.6 %). In contrast, CBT therapists treated a relatively larger proportion of patients with severe depression (33.3 %) compared to the overall practitioner mean proportion (28.2 %), PWPs (25.9 %), and counselors (27.4 %).

**Table 2:** Patient descriptives

Patient descriptive	PWP patients (n = 2153)		CBT therapist patients (n = 1199)		Counselor patients (n = 1628)		All patients (n = 4980)	
	N	%	N	%	N	%	N	%
Sex								
Male	745	34.6	485	40.5	402	24.7	1632	32.8
Female	1408	65.4	714	59.5	1222	75.1	3344	67.1
Age								
15–29	554	25.7	326	27.2	277	17.0	1157	23.2
30–49	1008	46.8	610	50.9	752	46.2	2370	47.6
50–69	523	24.3	253	21.1	534	32.8	1310	26.3
70–89	68	3.2	10	.8	65	4.0	143	2.9
Ethnicity								
White	1963	91.2	1043	87.0	1453	89.3	4459	89.5
Asian	72	3.3	40	3.3	51	3.1	163	3.3
Black	28	1.3	42	3.5	42	2.6	112	2.2
Mixed	48	2.2	34	2.8	30	1.8	112	2.2
Other	40	1.9	39	3.3	27	1.7	106	2.1
Employment								
Unemployed	574	26.7	451	37.6	492	30.2	1517	30.5
Not unemployed	1579	73.3	748	62.4	1112	68.3	3439	69.1
Depression (PHQ-9)								
Mild (5–9)	423	19.6	172	14.3	302	18.6	897	18.0
Mod (10–14)	570	26.5	295	24.6	421	25.9	1286	25.8
Mod sev (15–19)	603	28.0	333	27.8	459	28.2	1395	28.0
Sev (20–27)	557	25.9	399	33.3	446	27.4	1402	28.2
Functional impairment (WSAS)								
Subclinical (0–9)	353	16.4	118	9.8	336	20.6	807	16.2
Clinical								
Less sev (10–20)	978	45.4	435	36.3	700	43.0	2113	42.4
Mod sev to sev (21–40)	822	38.2	646	53.9	592	36.4	2060	41.4
Relative deprivation level (IMD)								
Low (0–25.00)	1138	52.9	546	45.5	621	38.1	2305	46.3
Mod (25.01–50.00)	635	29.5	392	32.7	609	37.4	1636	32.9
High (50.01–76.00)	379	17.6	258	21.5	393	24.1	1030	20.7

Across all patients, the majority were female (67.1 %) with a mean age of 41.7 years (SD = 14.0 years), of white ethnicity (89.5 %), and in some form of paid or unpaid task (69.4 %; that is, employed full-time or part-time, a homemaker, student, or retired). Most patients scored at clinical levels of impaired functioning (83.8 %) on the WSAS (Mundt et al. 2002; see measures section below), with fewer patients (20.7 %) living in relatively high deprivation geographical areas. The current study examined patients presenting with depression or comorbid depression and anxiety. Notably, patient depression (PHQ-9) and anxiety (GAD-7) showed a large significant positive correlation, *r* = .71, *p* = .000, 95 % CI [.69, .72].

### Measures

#### Practitioner Measures (Self Report)

##### *Connor*–*Davidson Resilience Scale (CD*-*RISC; Connor and Davidson*^2003^*)*

This is a 25-item measure incorporating the key characteristics of resilient people (Connor and Davidson 2003). These included hardiness, control, commitment, seeing change as a challenge (Kobasa 1979) and patience/perseverance through stress. The total CD-RISC scores range from 0 to 100. The CD-RISC has an internal consistency of .89, correlations between items range from .3 to .7 and the reported test–retest reliability is .87 (Connor and Davidson 2003).

##### *Mindful Attention Awareness Scale (MAAS; Brown and Ryan*^2003^*)*

This is a 15-item measure of mindfulness. The MAAS measures mindfulness as a trait and contains items designed to measure “an open or receptive attention to and awareness of on-going events and experience” (Brown and Ryan 2004; p. 245). The measure was designed to exclude attitudinal and motivational components, products (versus the process) of mindfulness, and items that implied refined levels of consciousness. Total MAAS scores range from 15 to 90. Contrary to usual reporting of average scores, the final raw score in the current study is expressed as a total score of all 15 items. The MAAS has an internal consistency ranging from .80 to .90 and a test–retest reliability of .81.

#### Patient-Completed Primary Outcome Measure

##### *Patient Health Questionnaire*-*9 (PHQ*-*9; Kroenke et al.*^2001^*; Spitzer et al.*^1999^*)*

The PHQ-9 is a brief (9-item) self-report measure of depression. Items request ratings of how often a person has been bothered by the various symptoms of depression over the previous 2-week period. Individual item scores range from 0 (“not at all”) to 3 (“Nearly every day”) with total PHQ-9 scores ranging from 0 to 27. Scores ≥ 10 indicate a clinical level of depression. Scores of 5–9, 10–14, 15–19, and 20–27 are classified as reflecting mild, moderate, moderately severe, and severe levels of depression respectively (Kroenke and Spitzer 2002). The measure has an internal reliability of .89 and a test–retest reliability of .84.

#### Secondary Measures Used in Multilevel Modeling

##### *Work and Social Adjustment Scale (WSAS: Marks*^1986^*; Mundt et al.*^2002^*)*

The WSAS is a 5-item self-report measure of functional impairment attributable to an identified psychological disorder, with items assessing five domains of functioning: work, home management, social-leisure activities, private-leisure activities, and relationships with others. Total WSAS scores range from 0 to 40. The measure has an internal reliability of .83 (Zahra et al. 2014) and a test–retest reliability of .73 (Mundt et al. 2002).

##### *Index of Multiple Deprivation*

Deprivation had been measured using the Index of Multiple Deprivation 2010 (IMD, Department for Communities and Local Government 2011). The IMD is an aggregation of multiple deprivation indices (including income, employment, health and disability, education, skills/training, barriers to housing and services, crime and living environment). The IMD identifies concentrations of geographical deprivation and can be used as a relative (as opposed to an absolute) measure of deprivation where higher IMD values reflect higher deprivation levels.

### Procedure

In the IAPT stepped care model, patients are assessed for depression, anxiety, and functioning by PWPs using the PHQ-9 (Spitzer et al. 1999), Generalised Anxiety Disorder-7 (GAD-7; Spitzer et al. 2006), and the WSAS (Mundt et al. 2002) respectively. Patient “caseness” or classification of severity is ascertained using PHQ-9, GAD-7, and clinical judgement. Patients with mild or moderate levels of depression and/or anxiety receive a low-intensity treatment from PWPs (Step 2) in the form of guided self-help. Those patients assessed as moderately severe or non-responsive to a Step 2 intervention are ‘stepped-up’ to receive traditional high intensity treatments of CBT or counseling (Step 3). Allocation of patients is also determined by other factors including patients’ treatment preferences (i.e., CBT or counseling treatment for high intensity treatment) and availability of practitioners.

### Treatment

Practitioners reported providing treatment consistent with their professional roles and personally identified with specific approaches consistent with their respective roles: PWPs’ responses included CBT, cognitive restructuring, problem solving and relaxation; CBT therapists’ responses included CBT, acceptance and commitment therapy, behavioral activation, and/or mindfulness; counselors’ responses included counseling for depression, person-centered therapy, emotion-focused therapy, psychodynamic therapy, and integrative approaches. Treatment duration ranged from 1 to 33 sessions, with a modal number of 1 session provided to 1848 patients (34.2 %) and a mean of 4 sessions (SD = 4.1). The average treatment duration was 2.5 sessions (SD = 2.2) for PWP interventions, 6.8 (SD = 5.3) for CBT and 5.0 (SD = 4.1) for counseling.

### Statistical Analyses

Preliminary analysis was conducted to determine whether practitioner groups significantly differed on each personal aspect, given the heterogeneous nature of the practitioner sample. Secondly, analyses central to the current study (i.e., benchmarking followed by multilevel modeling) were conducted, identifying significant differences in personal aspects between more and less effective practitioners. Finally, post hoc analyses examined the empirical role and contribution of the personal aspects.

The patient outcome dataset was analysed using MLwiN version 2.30 (Rasbash et al. 2009) and IBM SPSS Statistics version 21. MLwiN was applied to generate multilevel models with parameter values derived using the Iterative Generalised Least Squares (IGLS) estimation procedure. SPSS was used for all other analyses. Confidence intervals were derived using a web-based calculator of confidence intervals for correlations—how2stats (“how2stats,” 2015).

#### Single-Level Benchmarking Analysis

Aggregated practitioner-level distributions were derived in order to rank practitioners on their effectiveness using a patient index of change criterion, namely reliable improvement (Jacobson and Truax 1991). Two distributions for practitioners were established reflecting practitioners’ proportion of patients who showed reliable improvement for: (1) patients with mild to moderate depression only (5 ≤ PHQ ≤ 14), and (2) patients with moderately severe to severe depression only (PHQ ≥ 15). For each distribution, the lower quartile (i.e., lower 25 %) and upper quartile (i.e., upper 25 %) were used as benchmarks to identify less and more effective practitioners respectively. Comparisons of resilience and mindfulness between the less and more effective practitioner groups were conducted using independent samples t-tests.

#### Multilevel Modeling Analysis

A 2-level model (i.e., patients nested within practitioners) was generated taking patients PHQ-9 final session score as the dependent variable, with explanatory patient variables comprising pre-treatment PHQ-9 scores, employment status, ethnicity, functioning level, age, and deprivation level. The purpose of the model was to control for case-mix (patient variables) only when identifying more and less effective therapists. Putting therapist variables (e.g., practitioners’ age, experience, and professional roles) in the model and controlling for them would have had the effect of re-classifying more and less effective therapists based on their characteristics that cannot be changed. The model was hence able to identify more and less effective practitioners within the naturalistic practice-based setting, and identify findings comparable with those of the single-level analysis described above (i.e., whether practitioners are consistently more or less effective across both analyses). Using the final multilevel model, a residual plot—termed a *caterpillar plot*—was derived that reflected how practitioners varied in their patient outcomes against a population mean. The caterpillar plot comprised 95 % confidence intervals of patients’ final session outcome score residuals for each practitioner. Practitioners were grouped accordingly as more or less effective and comparisons of differences for resilience and mindfulness between these two groups were conducted using independent samples t-tests.

## Results

### Practitioner Personal Aspect Scores

Table 3 presents the means and standard deviations for resilience and mindfulness scores for PWPs, CBT therapists, and counselors. There was a significant difference between the professional groups for mindfulness, F(2, 34) = 3.35, *p* = .047 but not for resilience, F(2, 34) = 2.60, *p* = .090. For mindfulness, counselors scored significantly higher than PWPs (*t*(23) = −2.48, *p* = .021; counselors, M = 68.82, SD = 8.45; PWPs, M = 58.63, SD = 11.80). There were no significant differences in mindfulness between counselors and CBT therapists (*t*(27) = −1.33, *p* = .200), or between CBT therapists and PWPs, *t*(18) = 1.32, *p* = .204. There was a significant positive correlation between resilience and mindfulness for counselors, *r* = .61, *p* = .009, 95 % CI [.19, .85] but not for PWPs, *r* = .14, *p* = .75, or CBT therapists, *r* = .07, *p* = .82.

**Table 3:** Personal aspect scores across practitioner groupings

	Sample size	Resilience (R)		Mindfulness (M)	
		M	SD	M	SD
PWPs	8	63.13	11.37	58.63	11.80
CBT therapists	12	70.75	7.66	64.58	8.48
Counselors	17	71.76	8.93	68.82	8.45

**Fig. 1:**
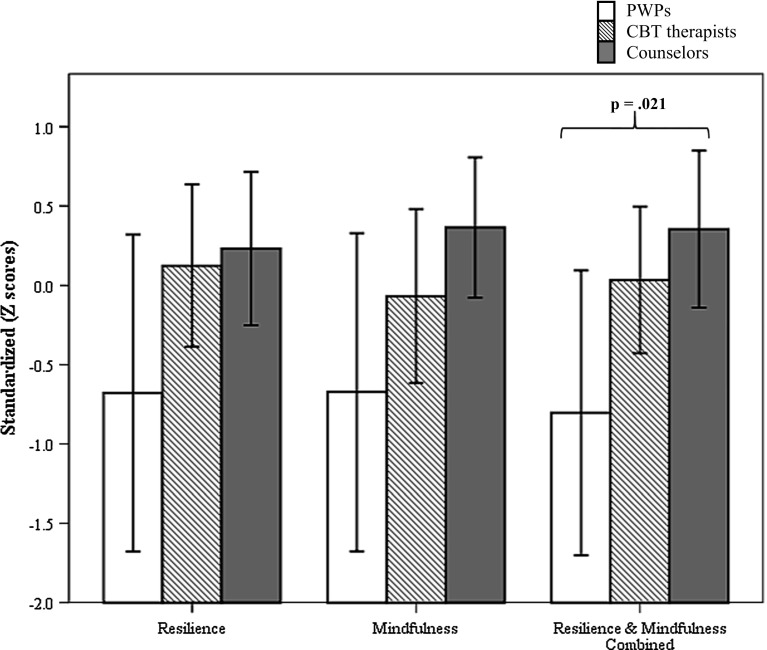
Resilience, mindfulness and resilience-mindfulness combined in PWPs, CBT therapists, and counselors

Figure 1 displays the standardized scores for resilience, mindfulness, as well as resilience and mindfulness combined for the three professional groups. Significant differences were evident between the professional roles on resilience and mindfulness combined, F(2, 34) = 4.36, *p* = .021. Three independent samples t-tests between practitioner pairs yielded a significant difference between counselors and PWPs, *t*(23) = −2.71, *p* = .013. Counselors scored significantly higher on resilience and mindfulness combined (M = .35, SD = .96) compared to PWPs (M = −.80, SD = 1.07). No significant difference was found between counselors and CBT therapists, *t*(27) = −.97, *p* = .340, while the difference between PWPs and CBT therapists approached significance, *t*(18) = 2.09, *p* = .051.

### Single-Level Benchmarking

Two sub-groups comprising more and less effective practitioners were identified for the treatment of patients with (a) mild to moderate depression, and (b) moderately severe to severe depression. These were practitioners who fell within the lower and upper quartiles of distributions of aggregated patient reductions in PHQ-9 scores. For patients with mild to moderate depression, the lower and upper quartiles both contained nine practitioners in each group (i.e., nine less effective practitioners and nine more effective practitioners). For patients with moderately severe to severe depression, there were 9 less effective and 10 more effective practitioners. Notably, six practitioners were consistently less effective and four consistently more effective when working with patients experiencing mild to moderate depression as well as with patients experiencing moderately severe to severe depression.

**Fig. 2:**
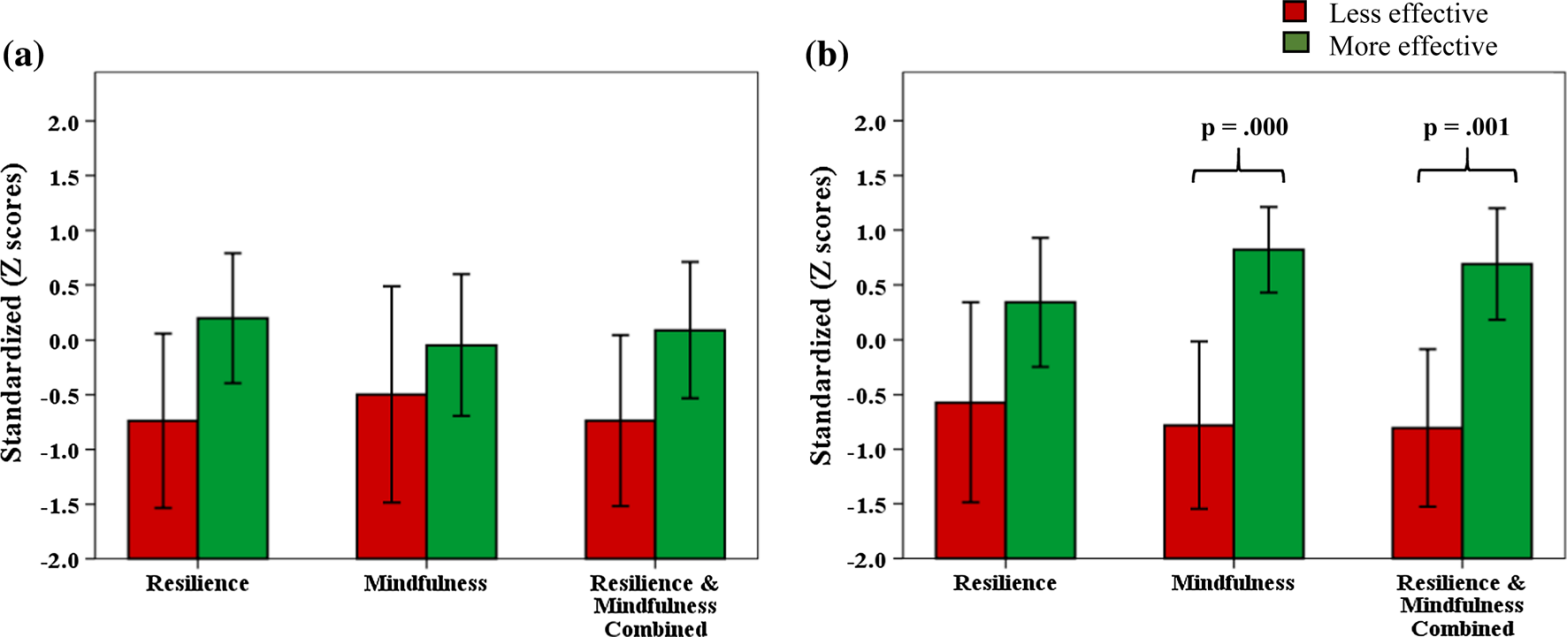
Personal aspect scores of less and more effective practitioners

Table 4 presents the mean standardized scores, standard deviations and t-test values for resilience, mindfulness, and resilience and mindfulness combined for less and more effective practitioner groups. A Bonferonni correction for the six comparisons (i.e., 2 patient severity groups × 3 personal aspect variables) yielded a significance criterion of *p* = .0083. The differences are presented graphically in Fig. 2. When working with mild to moderately depressed patients, there were no significant differences for resilience and mindfulness of practitioners either alone or combined: resilience, *t*(16) = 2.17, *p* = .045; mindfulness, *t*(16) = .89, *p* = .389; resilience and mindfulness combined, *t*(16) = 1.91, *p* = .075. When working with moderately severe to severely depressed patients, significant differences were evident for practitioner mindfulness, *t*(16) = 4.41, *p* = .000, as well as resilience and mindfulness combined, *t*(16) = 3.94, *p* = .001. No significant difference was obtained for resilience, *t*(16) = 1.97, *p* = .066.

**Table 4:** Personal aspect standardized scores of more and less effective practitioners according to patient depression severity

	Mild to moderate depression patients				Moderately severe to severe depression patients			
	Less effective (SD)	More effective (SD)	t-test value	t-test *p* value	Less effective (SD)	More effective (SD)	t-test value	t-test *p* value
Resilience	−.74 (1.04)	.20 (.77)	2.17	.045	−.57 (1.19)	.34 (.82)	1.91	.066
Mindfulness	−.50 (1.28)	−.05 (.84)	.89	.389	−.78 (1.00)	.82 (.55)	4.41	.000**
Resilience and mindfulness combined	−.74 (1.02)	.09 (.81)	1.91	.075	−.81 (.94)	.69 (.71)	3.94	.001**

* *p* < .0083; ** *p* < .0016

### Multilevel Modeling

Multilevel modeling was applied to the multilevel data where patients (Level 1) were nested within practitioners (Level 2). Initially, a conditional model containing only patient pre and post treatment PHQ-9 scores yielded an estimated therapist effect of 7.3 %. A single level model was then developed to control for pre-treatment PHQ-9 scores and patient case-mix. Five patient variables and the interaction of each patient variable with patients’ initial depression score were inserted into the model through a series of 10 stages. The final multilevel model, presented in the Appendix, comprised the dependent variable of post-treatment PHQ-9 scores, with explanatory variables comprising patient pre-treatment PHQ-9 scores, employment status, ethnicity, functioning, age, interaction between patient age and initial severity and deprivation level. After controlling for these patient characteristics, the therapist effect reduced to 6.7 %.

**Fig. 3:**
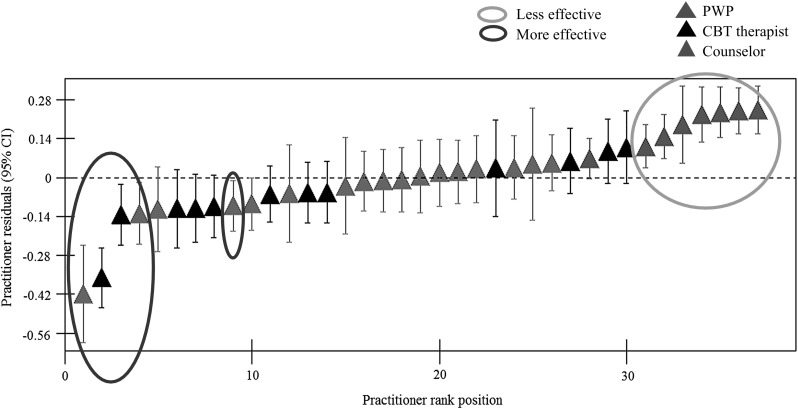
Residual plot of final multilevel model

Figure 3 presents a caterpillar plot based on the final model. Each vertical bar represents a practitioner, specifically a practitioner’s confidence interval of residual patient post-treatment depression scores. The plot identifies more effective practitioners (highlighted within the green circles) and less effective practitioners (highlighted within the red circle). The confidence intervals of more effective practitioners fall below and do not cross the dotted horizontal line, which represents the outcomes for the average practitioner (i.e., the post-therapy patient score is significantly less than the average post-therapy patient score). By comparison, the confidence intervals that are located above the overall practitioner mean and do not cross it represent less effective practitioners (i.e., where the post-therapy patient score is significantly greater than the average post-therapy patient score).

Figure 3 shows there to be 5 more effective, 25 effective, and 7 less effective practitioners. Relative to the high-intensity practitioners (i.e., CBT therapists and counselors), PWPs facilitated significantly less patient improvement. The more effective practitioners comprised 2 CBT therapists and 3 counselors, while a majority of the sample comprised effective practitioners (10 CBT therapists, 14 counselors and 1 PWP). Two of the more effective and 6 of the less effective practitioners in the MLM analyses were consistently identified as more and less effective in the benchmarking analysis (i.e., in both the treatment of patients with relatively milder and more severe levels of depression).

**Fig. 4:**
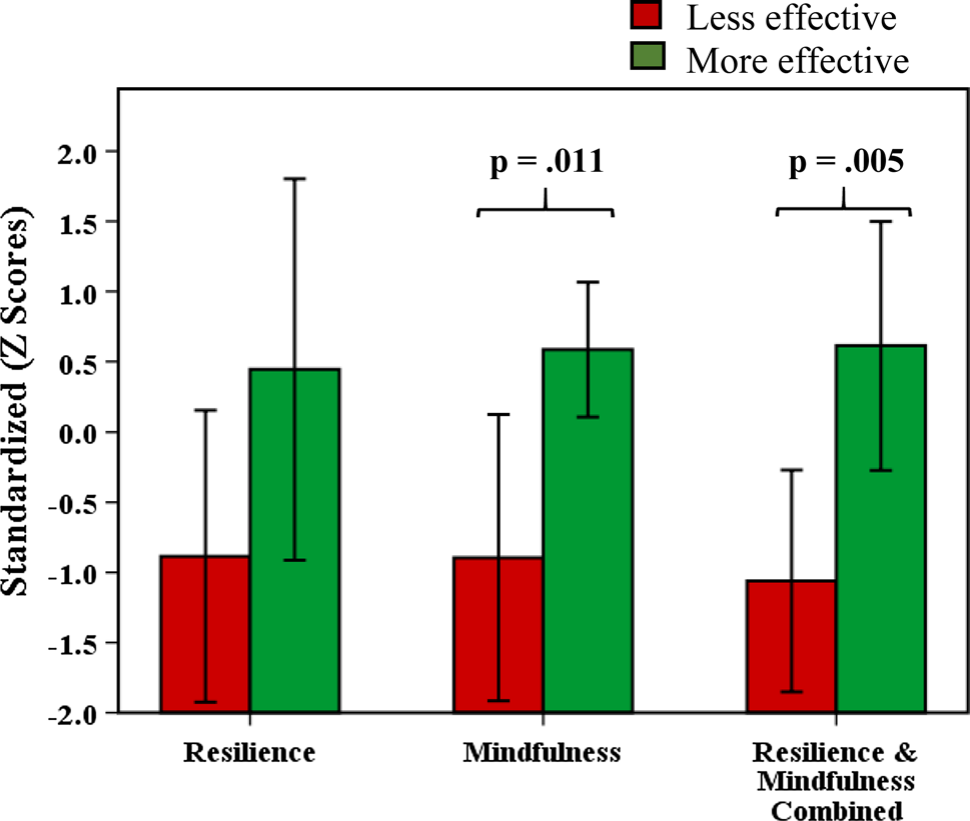
Mean personal aspect scores of less and more effective practitioners

**Fig. 5:**
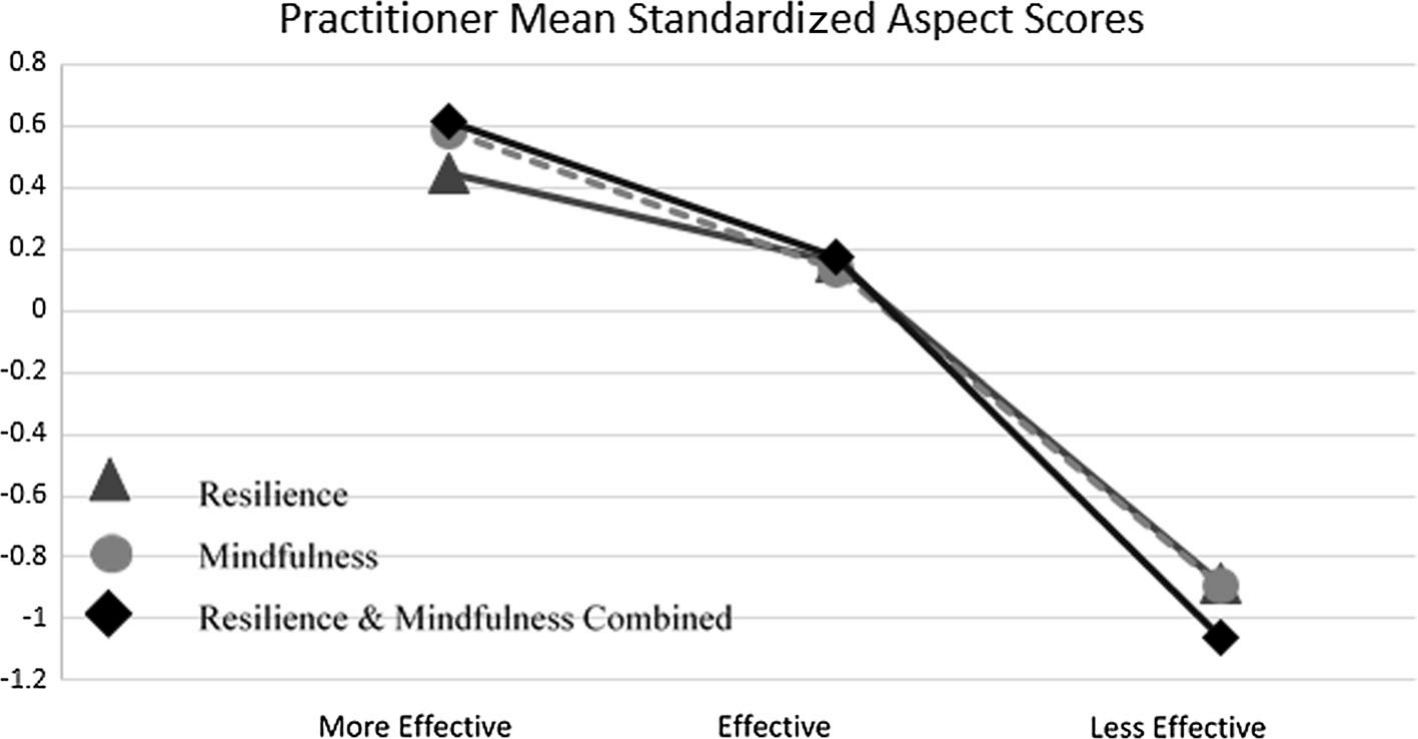
Mean standardized personal aspect scores for all practitioners

Table 5 and Fig. 4 depict the mean standardized personal aspect scores of the less and more effective practitioner groups and related t-test findings. Three independent samples t-tests were conducted adopting a Bonferroni correction of .017. Significant differences between less and more effective practitioners were found for mindfulness, *t*(10) = 3.29, *p* = .011, and resilience and mindfulness combined, *t*(10) = 3.57, *p* = .005. No significant difference was found for resilience, *t*(10) = 2.05, *p* = .068. Figure 5 extends the reporting to include all practitioners (i.e., including the effective practitioners) and shows a clear monotonic decrease in personal aspects from more effective to less effective practitioners.

**Table 5:** Personal aspect standardized scores of more and less effective practitioners

	Less effective (SD)	More effective (SD)	t-test value	t-test *p* value
Resilience	−.89 (1.12)	.44 (1.09)	2.05	.068
Mindfulness	−.90 (1.10)	.59 (.39)	3.29	.011*
Resilience and mindfulness combined	−1.06 (.85)	.61 (.71)	3.57	.005*

* *p* < .017; ** *p* < .003

### The Role and Contribution of Resilience and Mindfulness: Alone and Combined

Practitioner personal aspect variables were each inserted into the final multilevel model which controlled for patient characteristics. Significant contributions of these practitioner variables to patient outcome were then identified. Resilience, mindfulness, and resilience and mindfulness combined, of practitioners, each improved the final multilevel model. This was shown by the significant reduction in the −2LL ratio for resilience, χ^2^(1) = 6.43, *p* = .011, mindfulness χ^2^(1) = 6.64, *p* = .001, and resilience and mindfulness, χ^2^(1) = 9.79, *p* = .002.

Table 6 displays the relevant fixed and random model coefficients and the accompanying therapist effect values of the models examined. The reduction in therapist effect from 6.7 % was due to a decrease in practitioner variance related to the inclusion of the respective personal aspect or personal aspect combination into the model. Resilience and mindfulness separately contributed by a similar magnitude towards reducing patient outcome scores. These comprise *β* = −.067 (SD = .026) and *β* = −.068 (SD = .025) respectively. When resilience and mindfulness were both inserted as separate variables, both variables made no significant contribution towards patient outcome but reduced the therapist effect from 6.7 to 4.9 %. However, when resilience and mindfulness were inserted as a combined variable, a significant contribution was found, *β* = −.082 (SD = .024) with the same therapist effect value of 4.9 %.

**Table 6:** Personal aspect related fixed and variable coefficients in multilevel models

Final multilevel model with personal aspect	Contribution to outcome score (β) (fixed coefficient)	Practitioner variance (variable coefficient)	Therapist effect (%)
Resilience	−.067	.020	5.7
Mindfulness	−.068	.019	5.4
Separate resilience and mindfulness	(ns)	.017	4.9
Resilience and mindfulness combined	−.082	.017	4.9

## Discussion

The current study aimed to identify personal aspects that differentiated between more effective and less effective practice. Controlling for case-mix, a therapist effect of 6.7 % was found, which was partly explained by more effective practitioners having significantly higher levels of mindfulness alone as well as resilience and mindfulness combined when compared with less effective practitioners.

The finding for mindfulness alone, and resilience and mindfulness combined was robust given its consistency across (1) the different types of analyses conducted (i.e., traditional benchmarking and more adaptable multilevel modeling) and (2) differing groups of more and less effective practitioners. Looking across the differing groups, although more and less effective practitioners comprise differing individuals, these personal aspects are consistently associated with more effective practice. The role of practitioner mindfulness, as well as resilience and mindfulness combined, was evident in the effective treatment of patients with more severe levels of depression. In contrast, for less severely depressed patients, no statistically significant difference was found. Practitioner resilience for this patient group, however, was found to approach significance. These findings reiterate that practitioner (i.e., therapist) factors matter more when treating more severely depressed patients (Saxon and Barkham 2012).

When examining the personal aspects while controlling for patient variation, each personal aspect—resilience *or* mindfulness—was found to significantly improve patient outcomes. When practitioners treat an *average* patient, practitioners’ resilience or their mindfulness separately contribute by a comparable degree. However, when the contribution of resilience and mindfulness are considered in the same MLM analysis as separate entities, the contribution of each is no longer significant. By contrast, when combined additively, resilience *and* mindfulness significantly contribute to patient improvement by a relatively larger degree. This combined variable accounts for a relatively larger proportion of variance between practitioners compared to resilience or mindfulness alone.

One interpretation of these findings is that resilience and mindfulness have features that are incompatible and overlapping at the same time. The findings also suggest that the personal aspect combination constitutes a unique entity in itself that is greater than the sum of the separate personal aspects. While prior research suggests that the relationship between resilience and mindfulness pertains to individuals’ wellbeing, in the context of providing psychotherapy the overlapping features of resilience and mindfulness perhaps relate to practitioners’ resilience as informed by mindfulness. Referring to the current operationalization based on the measures used, the findings may relate to practitioners’ drive to maintain high standards, personal competence, and commitment to patients that is harnessed or guided by present moment observations that enable timely, congruent, and personalized therapeutic communication.

Although combined resilience and mindfulness were found to be higher amongst more effective individual practitioners of different theoretical orientations (i.e., CBT therapists and counseling), systematic differences between practitioner groups were identified. Collectively, a positive relationship between these personal aspects was only evident for counselors. This finding suggests that certain personal aspects may be conducive to particular professional identities, orientations, or philosophies. This raises two questions: How might different theoretical orientations influence practitioners’ personal aspects? And, how might practitioners, as individuals and regardless of their theoretical orientation, apply the combined personal aspects?

One understanding could be derived from considering group differences between the theoretical orientations and related professional socialisation. Counseling, by definition, is relatively flexible in its structure (i.e., a non-manualised approach, with less structured session formats). Counselors may feel more able to engage mindfully while working with patients given the lesser definitive structure of their approach. By contrast, the CBT approach of utilising session structuring and delivery of manualised disorder-specific treatment protocols could mean that therapist attention is focussed towards adherence that may reduce attentional capacity for mindful awareness. Maintaining close adherence to technique, attending to patient symptoms and possible reliance on procedural memory, may preclude mindful engagement with patients, with implications on treatment improvement, particularly for patients with more severe and complex depression (Stanley et al. 2006).

In respect to practitioners’ personal aspects at an individual level, CBT therapists and counselors did not significantly differ in their reported levels of resilience and mindfulness. Similarly no difference was identified for the combined personal aspect. These findings would suggest that high intensity practitioners possess comparable capacity to engage with patients in a resilient and/or mindful manner. It is possible that irrespective of the group effect described above, practitioners at an individual level could engage with patients while drawing on these personal aspects.

When considering PWPs together with counselors and CBT therapists, PWPs were identified as less effective relative to the high intensity practitioners. Notably PWPs reported significantly lower levels of mindfulness compared to counselors. This difference could be attributed to PWPs core training in providing brief time-limited protocol-driven interventions to large volumes of patients, as is consistent with the IAPT stepped care model. Due to the emphasis on provision of guided self-help by PWPs at Step 2, this may focus their attention in sessions and clinical supervision on adherence to treatment protocols. In the effort to provide rapid access to brief treatments in order to deliver patient turnover/throughput, they may by socialised to place much less reliance on mindfulness. The lower level of mindfulness may also be related to differences in practitioners’ ages between the groups of practitioners. Practitioners with more lived experience may find it relatively easier to remain in the present moment in the context of their more extensive life and work experiences.

### Research Limitations

We note three main limitations in the current study. First, findings may not generalize to the broader population of practitioners of psychological therapies. Specifically, the current findings are based on a modest, selective sample size of largely effective and more effective practitioners from the sample pool. Findings are further applicable to practitioners who work within a stepped care model (i.e., a systemized approach), who provide interventions for depression and anxiety, and whose patient depression outcome are scored on the PHQ-9. Second, the significant differences in personal aspects between more and less effective practitioners could be attributed to confounds within the sample and arising from the design of the study. These confounds relate to less effective practitioners (PWPs) who, are trained in providing less intensive treatment to mild-moderate patients, yet actually saw patients of broadly equivalent severity and a larger volume of patients. In addition, PWPs were systematically younger and hence less experienced than more effective practitioners. Third, in respect to the design of the study, historical patient data was examined. This made it impossible to monitor practitioners’ treatment application within the historical frame. However, all practitioners were trained in the IAPT model and received regular clinical supervision consistent with their respective treatment approach and therefore regarded as competent.

### Policy, Practice and Research Implications

The current findings identified systemic variability in practitioner effectiveness across professional roles. Less effective practitioners comprised those who saw patients with severity levels beyond their level of training. The findings call for policy makers to identify parameters associated with practitioners’ professional roles and to ensure that practitioners’ skills and training are matched with the severity levels of patients they see. Additionally, practitioners need to provide services within the remit of their respective professional roles.

Looking at the implications for practice and research, the findings suggest that practitioner effectiveness improves when practitioners utilise both mindfulness and resilience while working with more severely depressed patients. However, it could be argued that effective practice was more a function of practitioners’ age and experience rather than specific personal aspects of resilience and mindfulness. Research findings on the acquisition of expertise show that it is not experience per se but rather the quality and quantity (e.g., at least 10 years) of experience that contribute to becoming an expert (Ericsson et al. 1993). Mindfulness and resilience are suggested as components in this quality and quantity, respectively. Mindfulness is indicated through the quality of deliberate and conscientious practice (Barrick et al. 2001; Giluk 2009). Alternatively, resilience contributes to practitioners’ ability to persevere in deliberate practice over an extended time frame. Arguably, practitioners with relatively more mindfulness and resilience combined may readily accumulate deliberate experience that contributes to acquiring expertise in delivering effective practice. Older practitioners, with more life and work experience will have had more opportunity to engage in deliberate practice.

Direct implications relate to how these personal aspects can be enhanced through training and/or clinical supervision. Group differences suggest that a differential approach needs to be taken while respecting different theoretical orientations and professional roles. Training and/or clinical supervision could take into consideration the range of how practitioners personally utilise treatment (e.g., using procedural memory or more flexible application). Longitudinal therapist effects studies could evidence the role of staff training in cultivating mindfulness and resilience on patient outcomes over time.

In conclusion, the present study provides a test of the feasibility of how personal aspects of practitioners might be investigated in order to enhance our understanding of the variability that exists between practitioners and the impact this has on patient outcomes. Resilience and mindfulness are but two candidates. Future research needs to tap into a wider range of patient, therapist, procedural, relational factors and constructs to build a better understanding of the natural variability between therapists in order to inform policy makers. This will also enable intervention studies to be designed to test the ‘plasticity’ of therapist factors and their contribution to more effective patient outcomes.

## Appendix: Final Multilevel Model Which Controlled for Variability in Patient Pre-treatment Severity and Patient Case-Mix

$$\begin{aligned} {\text{LNphqLast}}_{ij} & = \beta_{0j} + 0.879(0.031)({\text{LNphqPre-gm}}_{{{ij} }}) + 0.160(0.051)({\text{LNphqPre-gm}}_{{{ij}}}^2) \\ & \quad + 0.148(0.019){\text{unemployed}}_{ij} + - 0.068(0.028){\text{white}}_{ij} + 0.005(0.001)({\text{WSASpre-gm}})_{ij} \\ & \quad + - 0.003(0.001)({\text{Age-gm}})_{ij} + - 0.003(0.001)({\text{Age-gm}}) \cdot ({\text{LNphqPre-gm}}_{{{ij} }}) \\ & \quad + 0.002(0.001)({\text{IMD-gm}})_{ij} + e_{ij} \\ \beta_{0j} & = 2.335(0.038) + u_{0j} \\ \end{aligned}$$

$$\begin{aligned} u_{0j} \sim {\text{N}}(0,\sigma_{u0}^{2} )\quad \sigma_{u0}^{2} = 0.024\;(0.006) \hfill \\ e_{ij} \sim {\text{N}}(0,\sigma_{e}^{2} )\quad \sigma_{e}^{2} = 0.332\;(0.007) \hfill \\ - 2 * loglikelihood = 8689.731\;(4965\;{\text{of}}\;4980\;{\text{cases}}\;in\;{\text{use}}) \hfill \\ \end{aligned}$$

*Note:* All continuous variables are centered around their grand means (gm). *LNphqLast*, *LNphqPre* are log-transformed patient post-treatment and pre-treatment outcome scores respectively. *Unemployed* and *White* are categorical variables refer to unemployed compared to employed patients and ethnically white compared to non-white patients. *WSAS* refers to patients’ degree of impaired functioning. *IMD* reflects patients’ degree of deprivation. *Age*-*gm.LNphqPre*-*gm* represent an interaction between patients’ age and patients’ pre-treatment scores.
